# Euphoric Presentation in Creutzfeldt-Jakob Disease and Its Diagnostic Implications: A Case Report

**DOI:** 10.7759/cureus.57419

**Published:** 2024-04-01

**Authors:** Mark Sahyouni, Bradley Casey, Zachary Carpenter, Frank Estrella, Chika Okafor

**Affiliations:** 1 Internal Medicine, Campbell University School of Osteopathic Medicine, Buies Creek, USA; 2 Internal Medicine, Cape Fear Valley Medical Center, Fayetteville, USA

**Keywords:** cerebrospinal fluid biomarker, 14-3-3 gamma, creutzfeldt-jakob disease, euphoria, cognitive deterioration, human prion disease

## Abstract

Creutzfeldt-Jakob disease (CJD) constitutes an aggressively advancing, terminal neurodegenerative condition classified within the spectrum of transmissible spongiform encephalopathies. The difficulty in establishing a diagnosis before death arises from the condition's rarity and the resulting limited level of suspicion attributed to it. The polymorphic nature of CJD symptoms contributes to the challenge of early diagnostic recognition. Emotional and behavioral changes have been well documented, but the initial presentation of euphoria has not been documented. Here, we present the case of a female patient who was experiencing an unusual state of euphoria followed by intermittently altered mental status. She was ultimately diagnosed with sporadic CJD, discharged home on hospice, and died within six months of discharge.

## Introduction

Creutzfeldt-Jakob disease (CJD) exemplifies a collection of infrequent neurological symptoms that can be hereditary and transmissible [1]. These symptoms are caused by prions-tiny, misfolded proteins that modify the structure of neighboring proteins, ultimately disrupting neuronal function [1]. Sporadic, genetic/familial, and acquired types are the three subgroups that comprise CJD [2]. The most prevalent form of CJD is sporadic, accounting for as much as 85% of cases [2]. Sporadic CJD causes abnormal folding of cellular prion protein (PrPc) from an alpha-helical structure to a PrPc scrapie (PrPSc) with an increased beta-pleated sheet structure [2]. The abnormal prions are determined to accumulate and spread quickly throughout the brain, converting adjacent, normally folded PrPc to PrPSc [2]. This progression is rapid, forming vacuoles, plaques, and a characteristic "spongy” appearance [2]. Pathological specimens portray severe neuronal loss, gliosis of the gray matter, and cerebral atrophy [2]. It tends to manifest between the ages of 55-75 years, with an average survival period ranging from four to eight months [2]. Although classified as an infection, CJD does not evoke the usual immune system or inflammatory response seen in most infectious diseases (i.e., fever, pain, edema, and/or leukocytosis, etc.), and the symptoms can vary [3]. Emotional and behavioral changes have been well documented in relevant literature, but the initial symptom of euphoria has not. We are introducing a compelling case involving a female patient who initially enjoyed her regular state of health but subsequently began experiencing the gradual emergence of numerous neurological symptoms that initially began with persistent euphoria.

## Case presentation

The case is a 61-year-old female with a past medical history of chronic anxiety who presented to the ED with her family for complaints of changes in her mentation. The family described it as an unusual state of euphoria with progressive memory loss for the last two months, which had become increasingly noticeable in the past three weeks. The patient stated she could not remember recent events or conversations, along with being uncharacteristically elated, which raised her family's suspicion. The family at the bedside reported that the patient would repeatedly tell everyone that she was "so happy" and would be laughing or smiling at socially incongruent scenarios.

When the patient was evaluated in the ED, she was extremely pleasant, confused, and wanted to stand in the corner of the room. On physical examination, the only abnormalities were her elated mood (despite her family's obvious concern for her health), memory, and orientation to person, place, and time; the patient was confused when answering questions, and her attention was mostly directed to looking out the window. Strength was 5/5 in all myotomes of bilateral upper and lower extremities, cerebellar function intact, sensation intact in all dermatomes in bilateral upper and lower extremities, and cranial nerves II-XII intact. Vital signs in the ED were grossly unremarkable. The patient's laboratory values during her ED visit can be found below (Table 1).

**Table 1:** Patient’s laboratory values during ED visit ED: emergency department

Laboratory Test	Patient’s Laboratory Value	Reference Range
Complete blood count		
White blood cell count	4.8 x 10*3/uL	4.5–12.5 x 10*3/uL
Hemoglobin	12.1 g/dL	12.0–16.0 g/dL
Mean corpuscular volume	86.0 fL	81.0–99.0 fL
Platelets	293 x 10*3/uL	150–450 x 10*3/uL
Comprehensive metabolic panel		
Sodium	139 mmol/L	136–145 mmol/L
Potassium (L)	3.3 mmol/L	3.5–5.1 mmol/L
Bicarbonate	27 mmol/L	21–32 mmol/L
Chloride	106 mmol/L	98–107 mmol/L
Blood urea nitrogen	17 mg/dL	7–25 mg/dL
Creatinine (L)	0.69 mg/dL	0.70–1.30 mg/dL
Glucose (H)	121 mg/dL	74–106 mg/dL
Aspartate aminotransferase	27 U/L	15–37 U/L
Alanine transaminase	27 U/L	12–78 U/L
Glomerular filtration rate	>60.0 mL/min/1.73m*2	>60.0 mL/min/1.73m*2
Albumin	4.5 g/dL	3.5–5.7 g/dL
Magnesium	2.1 mg/dL	1.9–2.7 mg/dL
Other blood tests		
Ethanol level	<3 mg/dL	<3 mg/dL
High sensitivity troponin	8 pg/mL	2–20 pg/mL
Three-hour high-sensitivity troponin	6 pg/mL	2–20 pg/mL
Rapid plasma reagin	Negative	Negative
Sedimentation rate (H)	26 mm/hr	0–15 mm/hr
C-reactive protein (H)	8 mg/L	<5 mg/L
Thyroid-stimulating hormone (H)	0.627 ulU/mL	0.450–5.330 ulU/mL
Hepatitis panel		
Hepatitis A antibody, immunoglobulin M	Negative	Negative
Hepatitis B surface antigen screen	Negative	Negative
Hepatitis B core antibody, immunoglobulin M	Negative	Negative
Hepatitis C antibody	<0.9 s/co ratio	0.0–0.9 s/co ratio
Human immunodeficiency virus screen 4th generation with reflex	Non-reactive	Non-reactive
Urine drug screen		
Benzodiazepine	Negative, none detected	Negative, none detected
Cocaine	Negative, none detected	Negative, none detected
Tetrahydrocannabinol	Negative, none detected	Negative, none detected
Phencyclidine	Negative, none detected	Negative, none detected
Amphetamine	Negative, none detected	Negative, none detected
Opiate	Negative, none detected	Negative, none detected
Methadone	Negative, none detected	Negative, none detected
Blood culture 1	Negative, no growth	Negative, no growth
Blood culture 2	Negative, no growth	Negative, no growth

**Figure 1:**
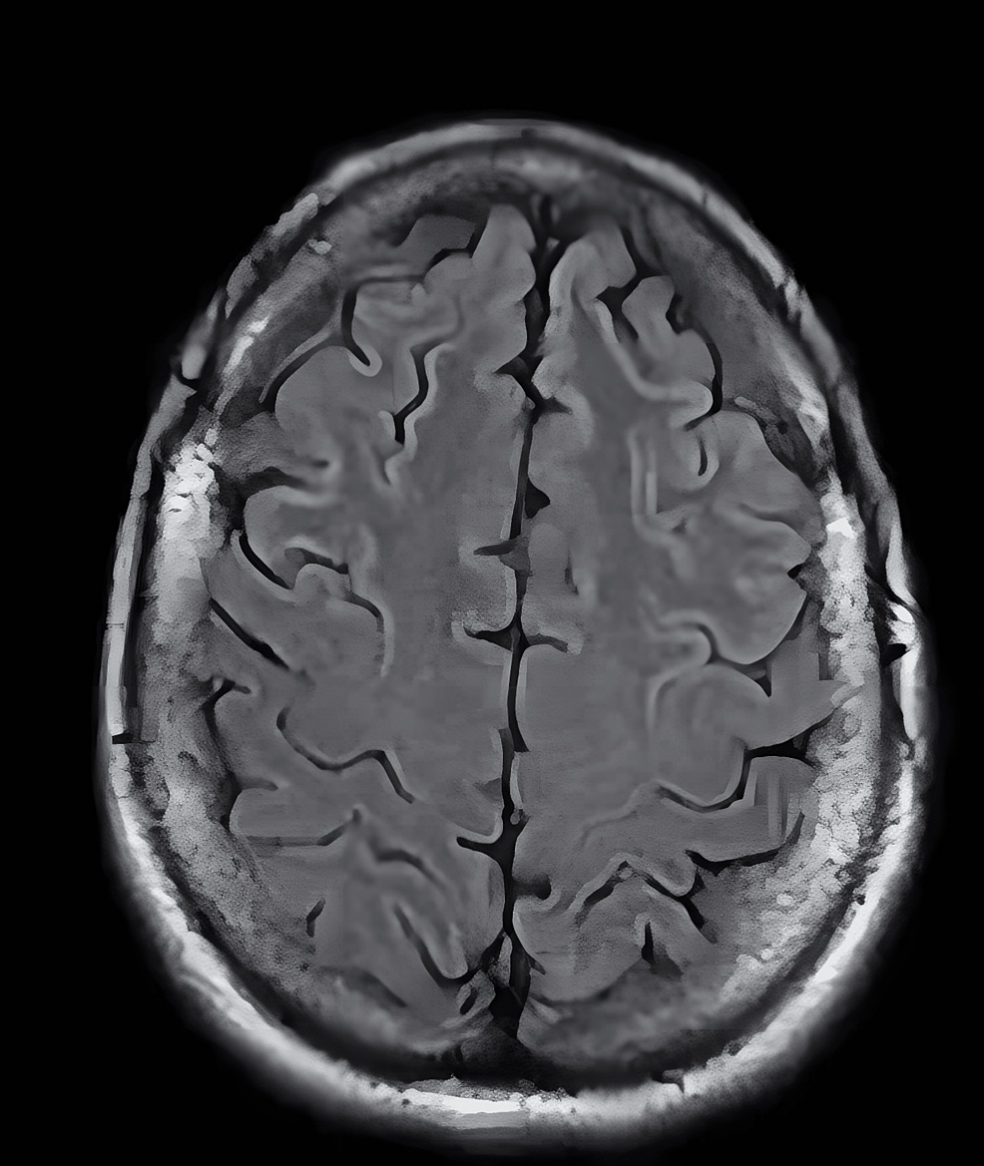
MRI of the brain reveals symmetric abnormal restricted diffusion and signal abnormality involving the cortex bilaterally MRI: magnetic resonance imaging

Due to the patient's altered mental status, a CT scan of the head was ordered, which revealed no acute abnormalities. The negative CT scan results prompted an order for an MRI of the head. The MRI showed symmetric abnormal restricted diffusion and signal abnormality involving the cortex bilaterally (Figure 1). Due to the abnormal MRI findings, a neurology consult was requested to evaluate the patient. The consulted neurologist ordered an EEG, but all attempts were unsuccessful due to patient non-compliance with the procedure. The neurologist was initially concerned about encephalitis/meningitis or CJD. A lumbar puncture was performed, and the encephalitis/meningitis panel was negative (Table 2). Cerebrospinal fluid (CSF) measurements of real-time quaking-induced conversion (RT-QuIC) came back positive (Table 2), total-tau protein and 14-3-3 gamma came back elevated (Table 2), all of which are diagnostic tests for CJD. The patient was officially diagnosed with sporadic CJD following interpretation of the results. Throughout the patient’s clinical course, she continued to decompensate with worsening memory, orientation to self, place, and time, and increased difficulty with activities of daily living. At the end of her hospital encounter, the family decided to take her home on hospice care, and she died within six months of discharge.

**Table 2:** Patient’s lumbar puncture results for encephalitis/meningitis and prion disease CSF: cerebrospinal fluid; RT-QuIC: real-time quaking-induced conversion

Diagnostic Tests	Patient’s Laboratory Value	Reference Range
Meningitis/encephalitis panel		
*Escherichia coli* K1 by polymerase chain reaction	Not detected	Not detected
*Haemophilus influenzae* by polymerase chain reaction	Not detected	Not detected
*Listeria monocytogenes* by polymerase chain reaction	Not detected	Not detected
*Neisseria meningitidis *by polymerase chain reaction	Not detected	Not detected
*Streptococcus agalactiae* by polymerase chain reaction	Not detected	Not detected
*Streptococcus pneumoniae* by polymerase chain reaction	Not detected	Not detected
Cytomegalovirus by polymerase chain reaction	Not detected	Not detected
Enterovirus by polymerase chain reaction	Not detected	Not detected
Herpes simplex virus 1 by polymerase chain reaction	Not detected	Not detected
Herpes simplex virus 2 by polymerase chain reaction	Not detected	Not detected
Herpes simplex virus 6 by polymerase chain reaction	Not detected	Not detected
Human parechovirus by polymerase chain reaction	Not detected	Not detected
Varicella zoster virus by polymerase chain reaction	Not detected	Not detected
*Cryptococcus neoformans/gattii* by polymerase chain reaction	Not detected	Not detected
Alpha-amino-3-hydroxy-5-methyl-4-isoxazolepropionic acid receptor antibody cytometric bead array	Negative	Negative
Amphiphysin antibody	Negative	<1:2
Anti-glial nuclear autoantibody-type 1	Negative	<1:2
Anti-neuron nuclear autoantibody-type 1	Negative	<1:2
Anti-neuron nuclear autoantibody-type 2	Negative	<1:2
Anti-neuron nuclear autoantibody-type 3	Negative	<1:2
Contactin-associated protein 2 immunoglobulin G cytometric bead array	Negative	Negative
Collapsin response mediator proteins 5 immunoglobulin G	Negative	<1:2
Dipeptidyl peptidase-like protein 6 antibody immunofluorescence assay	Negative	Negative
Gamma-aminobutyric acid type B antibody cytometric bead assay	Negative	Negative
Glutamic acid decarboxylase 65 antibody assay	0.00 nmol/L	<0.02 nmol/L
Glial fibrillary acidic protein immunofluorescence assay	Negative	Negative
Anti-immunoglobulin-like cell adhesion molecule 5 immunofluorescence assay	Negative	Negative
Leucine-rich glioma-inactivated 1 immunoglobulin G cytometric bead array	Negative	Negative
Metabotropic glutamate receptor 1 antibody immunofluorescence assay	Negative	Negative
Neuronal intermediate filament	Negative	Negative
N-methyl-D-aspartate receptor antibody cytometric bead array	Negative	Negative
Purkinje cell cytoplasmic antibody-TR	Negative	<1:2
Purkinje cell cytoplasmic antibody-1	Negative	<1:2
Purkinje cell cytoplasmic antibody-2	Negative	<1:2
Prion disease laboratory results		
CSF RT-QuIC (!)	Positive	Negative
Total tau proteins (H)	>20,000 pg/mL	0–1,149 pg/mL
14-3-3 gamma (H)	104,860 AU/mL	<30–1,999 AU/mL
Lumbar puncture (tubes 1 and 4)		
Appearance	Clear	Variable
Color	Colorless	Variable
Xanthochromia	Absent	Absent
White blood cell count	<3 cells/uL	0-5 cells/uL
Red blood cell count (H)	16 cells/mm*3	0-5 cells/mm*3
Total count, CSF	<3 cells/uL	0-5 cells/uL
Mononuclear %	100%	
Mononuclear #	1 cells/uL	
Polynuclear %	0%	
Protein (H)	60 mg/dL	15-45 mg/dL
Glucose (H)	71 mg/dL	40-70 mg/dL

## Discussion

CJD has been described since the early 1920s [4]. As stated by the Centers for Disease Control and Prevention, the likelihood of developing CJD rises as individuals grow older [4]. Over the period from 2016 to 2020, the incidence and prevalence in the United States were approximately five cases per million individuals aged 55 years or older [4]. Note that incidence and prevalence will be effectively the same due to expected mortality within one year of diagnosis. In the later phases of sporadic CJD, there is a striking resemblance in the physical presentation of all prion diseases [5]. The conventional manifestations of sporadic CJD encompass profound cognitive deterioration, cerebellar ataxia, myoclonus, and disruptions in myotomal functioning [5]. Typically, the initial symptoms of sporadic CJD are general and lack specificity, such as headache, fatigue, cough, dizziness, and alterations in personality, mood, or memory [5]. With the progression of the disease, patients have a characteristic myoclonus following a loud sound, along with being akinetically mute in advanced stages [4,6]. Emotional lability and changes in one's behavior have been well documented, but not euphoria.

CJD manifests a fatality rate of 100%, thus rendering the process of diagnosis of paramount significance [7]. While histopathological affirmation stands as the established benchmark for a conclusive diagnosis, innovative tests are now offering avenues for post-mortem identification that are comparably less intrusive than cerebral biopsy [7]. The diagnostic criterion without histopathology is difficult and includes the presence of a neuropsychiatric disorder concomitant with a positive result on the RT-QuIC test conducted on CSF [8]. Alternatively, a diagnosis of CJD can be established when dementia is observed along with at least two of the ensuing four clinical manifestations: myoclonus, indications of visual and/or cerebellar dysfunction, signs indicative of pyramidal and/or extrapyramidal involvement, and the state of akinetic mutism [8]. Furthermore, weighted MRI is a form of imaging that can be used to detect certain diseases. A study by Park et al. found that changes can be detected in either the thalami, basal ganglia, or cerebral cortex with a 91% sensitivity and 97% specificity for the disease [9]. Another diagnostic method is a CSF analysis yielding 14-3-3 protein [10]. Although this may show up in other pathologies, it has been found to have a sensitivity and specificity of 95-98% when used appropriately with clinical suspicion for CJD [10]. Lastly, the newest convention for diagnosing CJD is via RT-QuIC [11]. This method utilizes recombinant normally folded prion proteins and is then incubated with a sample of a patient with suspected CJD [11]. The samples are allowed to interact, and if positive, PrPSc should be amplified in the sample because of the seeding and conversion of PrPc to PrPSc [11]. RT-QuIC is 96% sensitive and 100% specific [11].

A retrospective medical record review by Paterson et al. investigated the misdiagnosis of patients with CJD and the differential diagnoses they were given prior to appropriate detection. Of the 97 patients that had proven CJD via cerebral biopsy, there were a total of 397 diagnoses given before CJD was properly diagnosed; these included diagnoses given by primary care physicians and neurologists [12]. Only 18% of patients were correctly diagnosed at their first assessment, usually by a neurologist [12]. The top five misdiagnoses given were viral encephalitis, paraneoplastic disorder, depression, peripheral vertigo, and Alzheimer's disease [12]. For all the patients, the average time for proper diagnosis was nearly eight months, which is an average of 66.7% of their disease course [12].

The treatment for CJD remains elusive to this day [2]. The cornerstone of management revolves around alleviating symptoms and providing comprehensive support [2]. While several pharmacological investigations pertaining to CJD have been undertaken, none have yielded discernible advantages [2]. Due to the rapid progression of the disease and 100% mortality, initiating arrangements for hospice care should be a priority for the healthcare team [2]. Providing counseling to the family should also be a priority for the multidisciplinary team upon discharge [2]. The orchestration of interprofessional efforts can contribute to a more seamless progression through the terminal stages of this ailment, involving comprehensive family guidance and the administration of suitable palliative measures [2].

## Conclusions

The presented case focuses on the initial atypical manifestation of euphoria that led to a diagnosis of sporadic CJD. The infrequency of such a presentation emphasizes the attentiveness required among providers. This case shows the significance of a complete neurological examination, imaging techniques, laboratory interpretations, and a multidisciplinary approach to unraveling the complexities of early-stage CJD diagnosis. This case serves as a reminder to keep a broad initial differential when a patient presents with unusual symptomatology. Research also supports the use of neurology consultation in patients with an unusual and otherwise unknown etiology of varying emotional presentation and cognitive impairment.
